# Renal thrombotic microangiopathy and podocytopathy associated with the use of carfilzomib in a patient with multiple myeloma

**DOI:** 10.1186/1471-2369-15-156

**Published:** 2014-09-30

**Authors:** Liliane Hobeika, Sally E Self, Juan Carlos Q Velez

**Affiliations:** Division of Nephrology and Hypertension, Department of Medicine, University of Louisville, 615 South Preston Street, Louisville, KY 40202 USA; Department of Pathology, Medical University of South Carolina, Charleston, SC USA; Division of Nephrology, Department of Medicine, Medical University of South Carolina, Charleston, SC USA

**Keywords:** Thrombotic microangiopathy, Malignant hypertension, Proteasome inhibitor, Proteinuria

## Abstract

**Background:**

Proteasome inhibitors are a relatively new class of chemotherapeutic agents. Bortezomib is the first agent of this class and is currently being used for the treatment of multiple myeloma. However, recent reports have linked exposure to bortezomib with the development of thrombotic microangiopathy. A new agent in this class, carfilzomib, has been recently introduced as alternative therapy for relapsing and refractory multiple myeloma. We report a case of renal thrombotic microangiopathy associated with the use of carfilzomib in a patient with refractory multiple myeloma.

**Case presentation:**

A 62 year-old Caucasian man with hypertension and a 4-year history of multiple myeloma, had been previously treated with lenalidomide, bortezomib and two autologous hematopoietic stem cell transplants. After the second hematopoietic stem cell transplant, he developed acute kidney injury secondary to septic shock and required dialysis for 4 weeks. Subsequently, his serum creatinine stabilized at 2.1 mg/dL (185.64 μmol/L). Seventeen months after the second hematopoietic stem cell transplant, he was initiated on carfilzomib for relapse of multiple myeloma. Six weeks later, he developed abrupt worsening of lower extremity edema and hypertension, and new onset proteinuria. His kidney function remained stable. Kidney biopsy findings were consistent with thrombotic microangiopathy. Eight weeks after discontinuation of carfilzomib, proteinuria and hypertension improved. Due to progression of multiple myeloma, he died a few months later.

**Conclusion:**

In view of the previously reported association of bortezomib with thrombotic microangiopathy, the temporal association of the clinical picture with the initiation of carfilzomib, and the partial resolution of symptoms after discontinuation of the drug, we conclude that carfilzomib may have precipitated a case of clinically evident renal thrombotic microangiopathy in our patient.

## Background

Because impairment of kidney function in patients with multiple myeloma (MM) can be caused by a variety of conditions, ascertaining the etiology of kidney dysfunction in patients with MM represents a challenging task for the practicing nephrologist. Patients with MM are at risk of acquiring acute kidney injury (AKI) as a result of light chain cast nephropathy [1], hypercalcemia [2], bisphosphonate-induced tubular injury [3] and lenalidomide nephrotoxicity [4]. Similarly, syndromes of glomerular involvement can also occur in MM as a result of light or heavy chain deposition disease, amyloidosis or bisphosphonate-induced podocytopathy. Furthermore, patients with MM who undergo hematopoietic stem cell transplantation (HSCT) are also at risk of acquiring renal syndromes inherent to HSCT, such as ischemic acute tubular necrosis and thrombotic microangiopathy (TMA) [5, 6]. The clinical features of TMA syndromes include microangiopathic hemolytic anemia, thrombocytopenia, and organ injury. The pathological features are vascular damage manifested by arteriolar and capillary thrombosis with characteristic abnormalities in the endothelium and vessel wall. Renal pathology in TMA is characterized by thickened capillary walls, occlusion of vascular lumens, fibrin deposition and endothelial separation with expansion of subendothelial zone.

Over the last few years, multiple reports have unveiled an association between anti-angiogenic therapy and TMA. Antineoplastic drugs designed to target vascular endothelial growth factor (VEGF) such as sunitinib, sorafenib, bevacizumab, and others, have been linked to the development of a syndrome characterized by severe hypertension and/or acute or chronic kidney injury, with or without proteinuria, and associated with histopathological evidence of TMA in the kidney [7, 8]. Bortezomib is a proteasome inhibitor that was approved by the Food and Drug Administration (FDA) in 2003 for the treatment of refractory MM and subsequently in 2008 as an initial treatment of patients with MM. Although it does not target VEGF directly, bortezomib has also been reported to be associated with TMA. In July 2012, a new member in its class, carfilzomib, was approved by the FDA for the treatment of relapsing or refractory MM.

In this report, we summarize the case of a patient with MM status post autologous HSCT and chronic kidney disease who experienced worsening hypertension along with a substantial increase in proteinuria shortly after the initiation of carfilzomib for the treatment of refractory disease. We propose carfilzomib as a possible trigger of malignant hypertension and renal TMA in this case.

## Case presentation

The patient was a 62 year-old Caucasian man with a long-standing history of essential hypertension and a 4-year history of MM (IgG kappa subtype). The latter was diagnosed after suffering a T7 compression fracture. At that time, his kidney function was normal (serum creatinine: 0.9 mg/dL (79.56 μmol/L)) and his blood pressure was fairly well controlled on four agents (carvedilol extended-release 80 mg daily, diltiazem 60 mg three times daily, valsartan 320 mg daily and hydralazine 25 mg three times daily). As initial therapy for MM, he received melphalan for conditioning, four cycles of lenalidomide and dexamethasone, followed by autologous HSCT. Three months later, his kidney function remained within normal limits. He subsequently developed a few episodes of volume depletion associated with transient increases in serum creatinine level, after which his serum creatinine stabilized at a level of 1.4 mg/dL (123.76 μmol/L). Ten months after HSCT, he was started on bortezomib, cyclophosphamide and dexamethasone (VCD) due to progression of MM. He received five cycles of VCD. His blood pressure remained fairly well controlled with no changes to his anti-hypertensive regimen. Fifteen months after the first HSCT and 1 month after completing VCD, he underwent a second autologous HSCT for relapse. This time, the hospital course was complicated with septic shock and a severe bout of AKI, with serum creatinine peaking at 7.4 mg/dL (654.16 μmol/L). After requiring four weeks of acute hemodialysis, he partially regained kidney function, ultimately being discharged from the hospital with a new baseline serum creatinine of 2.1 mg/dL (185.64 μmol/L). At the time of discharge, his antihypertensive regimen was modified to avoid blockade of the renin-angiotensin system in the setting of AKI. Accordingly, he was switched to hydralazine 50 mg three times daily, diltiazem extended-release 360 mg daily, metoprolol 200 mg twice daily and a clonidine patch 0.3 mg/24 h.

**Figure 1:**
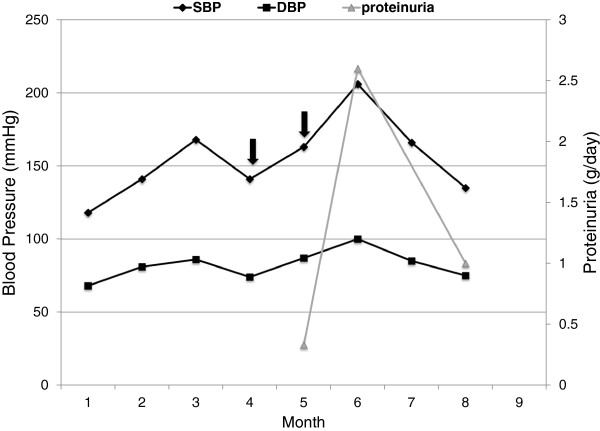
**Schematic illustrating the clinical and laboratory presentation of our patient.** Proteinuria in month 5 was measured on a spot urine sample. Proteinuria in months 6 and 8 was estimated from a 24-hour urine collection. Black arrows reflect carfilzomib administration (20 mg/m2 in month 4, and 27 mg/m2 in month 5).

Seventeen months after the second HSCT, a follow-up bone marrow biopsy specimen revealed persistent plasma cell infiltration. As a result, he was initiated on carfilzomib (20 mg/m^2^; followed by 27 mg/m^2^, four weeks apart), thalidomide 100 mg daily and dexamethasone 20 mg per week. Six weeks after the initiation of chemotherapy, the patient developed abrupt worsening of lower extremity edema and his hypertension became more difficult to control. After being stable with four agents (hydralazine, diltiazem, metoprolol and clonidine) averaging a blood pressure of 142/74 mmHg during previous office visits, he presented with a blood pressure of 206/100 mmHg (Figure 1). His physical examination also revealed pallor, but otherwise no additional abnormalities. Laboratory data showed: hemoglobin 8.2 g/dL (5.09 μmol/L), platelet count 53 K/cumm, serum creatinine 2.1 mg/dL (185.64 μmol/L), lactate dehydrogenase 183 IU/L, haptoglobin 23 mg/dL (0.23 g/L), total bilirubin 0.6 mg/dL (10.26 μmol/L), C3 112.3 mg/dL (1.123 g/L) (normal range: 88–201 mg/dL), C4 54.4 mg/dL (0.544 g/L) (normal range: 16–47 mg/dL), albumin 2.8 g/dL (28 g/L), serum kappa free light chain 82.4 mg/dL (normal range: 0.33-1.94), serum lambda free light chain 0.69 mg/dL (normal range: 0.57-2.63), serum free kappa/lambda ratio 119.42 (normal range: 0.26-1.65). Urinalysis showed 300 mg/dL protein on dipstick but no hematuria or pyuria. Urine protein electrophoresis showed elevated kappa light chain in the gamma zone at 11.9 mg/dL (232 mg per 24 hours) and a 24-hour urine collection revealed 2.6 grams of protein (53.3% albumin). Previous testing had shown presence of low-grade proteinuria (less than 0.5 grams per day) (Table 1). In addition to his antihypertensives, he was also taking acyclovir, citalopram, esomeprazole, zolpidem, tramadol and aspirin. He required the addition of eplerenone 25 mg daily, nifedipine extended-release 90 mg daily and benazepril 30 mg daily, for a total of 7 antihypertensives. Despite the 7-drug combination, he remained hypertensive averaging 190/95 mmHg.

**Figure 2:**
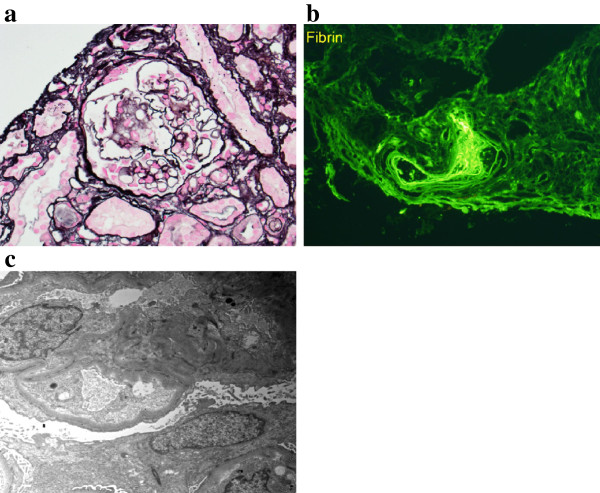
**Renal histologic findings in the patient. a**. Light microscopy: mesangiolysis on Jones’ methenamine silver stain. **b**. Immunofluorescence: Small artery intensely stained for fibrin. **c**. Electron microscopy: Endothelial cell swelling and flocculent material between endothelial cell and glomerular basement membrane.

A renal sonogram revealed a right kidney of 10.8 and a left kidney of 10.6 cm of longitudinal diameter with moderately increased cortical echogenicity. Following acute reduction of blood pressure with intravenous labetalol, an ultrasound-guided percutaneous kidney biopsy was performed to evaluate the newly developed overt proteinuria. Histological examination of the biopsy specimen on light microscopy disclosed 8 out of 17 globally sclerotic glomeruli, some mesangiolysis (Figure 2a), moderate interstitial fibrosis and tubular atrophy, and severe arteriolar hyalinosis. There was no evidence of myeloma cast nephropathy. No thrombi were identified in the glomerular capillaries or arterioles. Congo red stain was negative. Immunofluorescence showed 4 out of 14 glomeruli with global sclerosis and one small artery that stained intensely for fibrin (Figure 2b), C1q, IgM and C3. The corresponding H&E-stained cryosection showed a thrombus in that artery. The specimen was negative for linear deposition of IgG or kappa along the glomerular and tubular basement membranes. Electron microscopy of one glomerulus showed diffuse foot process effacement, endothelial cell swelling and some loops with flocculent material between the endothelial cell and the glomerular basement membrane (Figure 2c). There was no immune complex deposition or finely granular electron dense deposits along the glomerular or tubular basement membranes. In summary, the findings were consistent with TMA, glomerular podocytopathy, hypertensive-related injury and chronic scarring.

**Table 1:** **Laboratory values**

Variable (normal range)	2 months prior to biopsy	At time of biopsy	2 months after biopsy
Hemoglobin (14–18 g/dL)	8.8	8.2	8.0
Platelets (140–440 k/cumm)	75	53	62
Serum Creatinine (0.6-1.3 mg/dL)	2.1	2.1	1.7
LDH (100–240 IU/L)	NA	183	174
Haptoglobin (36–195 mg/dL)	NA	23	5
Serum M-spike (g/dL)	1.06	0.78	1.18
Proteinuria (0–0.3 g/day)	0.33	2.6	1.0
albumin on UPEP (%)	NA	53.3	49.3
M-spike on UPEP (%)	NA	8.8	13.7

Abbreviations: LDH, lactate dehydrogenase; NA, not available**;** UPEP, urine protein electrophoresis.

After the results of the kidney biopsy were reviewed and discussed, carfilzomib was discontinued. Eight weeks later, proteinuria slightly improved to 1 gram on a 24 hour urine collection. His serum creatinine remained stable at 1.7 mg/dL (150.28 μmol/L) at that time. His arterial blood pressure improved significantly averaging 135/75 mmHg on 5 agents. Due to progression of MM and a joint decision of not pursuing further treatment, the patient died four months later.

## Discussion

We present a case of an individual who experienced abrupt worsening of hypertension and proteinuria 6 weeks after receiving carfilzomib for the treatment of refractory MM. A kidney biopsy specimen revealed a TMA lesion along with podocytopathy and evidence of chronic scarring. No previous report of renal TMA associated with carfilzomib was found in published literature. Applying the Naranjo criteria for adverse drug reactions [9], the present case meets the criteria of ‘possible’ association with TMA. First, the timing of the administration of the drug and the subsequent clinical syndrome support our contention. The onset of worsening hypertension and proteinuria coincided with the initiation of carfilzomib, as depicted in Figure 1. Secondly, there is a precedent of reports of a drug of a similar class being associated with TMA. Thirdly, the patient partially recovered after the drug was discontinued.

Renal TMA is a known complication of HSCT that typically occurs approximately 3 months post-transplantation (range 14 – 240 days) [10]. Notably, it is much more likely to occur after allogeneic compared to autologous HSCT, occurring in 8-12% following allogeneic HSCT. Although factors inherent to HSCT, such as graft-versus-host disease, high-dose chemotherapy, and total body irradiation have been implicated in the pathogenesis of allogeneic HSCT-associated renal TMA, it is generally thought that the use of calcineurin inhibitors (CI) may largely explain the increased incidence of renal TMA [5, 6] after allogeneic HSCT given the well-described association between CIs and TMA in solid organ transplantation [11]. On the other hand, TMA associated with autologous HSCT is extremely rare, only case reports were found in the literature [12–14]. In our case, the second autologous HSCT was performed more than a year prior to the onset of worsening proteinuria and malignant hypertension. Therefore, the type of HSCT, the absence of CI therapy and the timing of the clinical presentation do not favor autologous HSCT as the primary etiology of renal TMA in our patient. Radiation therapy was not given in this case.

The development of overt proteinuria along with uncontrolled hypertension stimulated the clinical decision to perform a kidney biopsy. In addition to the TMA lesion, the biopsy specimen revealed evidence of podocytopathy in the form of diffuse foot process effacement, thereby explaining the proteinuric nature of the renal syndrome. Various degrees of focal or diffuse foot process effacement were reported in all the patients included in a case series describing the association of TMA and chemotherapy with monoclonal antibodies against vascular endothelial growth factor (VEGF) [8].

We identified 4 cases of bortezomib-associated TMA in the medical literature. Two of those reports described cases of patients with MM who developed microangiopathic hemolytic anemia and thrombocytopenia during the course of the first cycle of bortezomib [15, 16]. ADAMTS13 activity was normal in both cases. The subjects improved after drug discontinuation. Two additional reports described cases of microangiopathic hemolytic anemia and thrombocytopenia complicated with AKI [17, 18]. Although kidney biopsy was not performed, a renal TMA lesion was suspected. In addition to drug discontinuation, those patients underwent plasmapheresis.

Bortezomib is a dipeptide boronate 20S proteasome inhibitor. It inhibits nuclear factor kappa B (NF-KB) translocation/transcription activity by blocking the degradation of its inhibitor iKB. Inhibition of NF-KB leads to a decrease in VEGF transcription [19]. Production of VEGF by podocytes is indispensable for maintenance of the adjacent glomerular endothelium. Anti-VEGF chemoagents, such as bevacizumab and sorafenib, can cause TMA by direct microvascular toxicity initiated by endothelial injury [8, 20, 21]. Bortezomib can also inhibit IL-6 induced proliferation and angiogenesis and enhance the release of pro-inflammatory cytokines (IL-6 and TNF-alpha) and generation of cell mediated immune response [22]. In addition, it has been shown that VEGF, acting through VEGFR-2, activates endothelial nitric oxide synthase (eNOS) activity, leading to increased vasodilatory nitric oxide production. Anti-VEGF therapies might cause systemic deficiency of nitric oxide with consequent hypertension [23, 24]. Therefore, it has been speculated that the mechanism by which proteasome inhibitors may induce TMA also involve modulation of VEGF. Notably, although bortezomib is being increasingly used to treat antibody-mediated rejection in organ transplantation, no reports of associated TMA have emerged to date [25–27]. Therefore, the strength of the association requires further validation.

Carfilzomib is a tetrapeptide epoxyketone proteasome inhibitor. In preclinical studies, carfilzomib showed greater selectivity than bortezomib for the proteasome and had anti-proliferative activity in cells resistant to bortezomib [28]. In view of the previously reported association of bortezomib with TMA [15–18], we speculate that carfilzomib could have been involved in the pathogenesis of renal TMA in our case. In contrast to previous reports of proteasome inhibitor-associated TMA [15–18], our case describes a patient who presented with overt proteinuria and worsening hypertension but without extrarenal features of microangiopathic hemolytic anemia, i.e., renal-limited TMA. Undoubtedly, it could be argued that the long-standing history of essential hypertension could be responsible of some of the histological findings in the kidney biopsy specimen knowing that the renal TMA lesion in our patient is similar to that of malignant hypertension. Nonetheless, we hypothesize that the abruptness of the clinical presentation may reflect the onset of a mechanism of injury induced by carfilzomib, triggering malignant hypertension in a patient who was perhaps at high risk of developing it due to his underlying essential hypertension.

## Conclusions

In summary, we suggest that clinicians should be aware of the possibility of an association between exposure to carfilzomib and the development of a clinical syndrome characterized by worsening proteinuria and uncontrolled HTN and pathological evidence of renal TMA. Discontinuation of the drug should be considered only after careful evaluation of risks and benefits of the chemotherapy and the prognosis of the existing malignancy.

## Consent

Informed consent was obtained from the next of kin for publication of this case.

## Abbreviations

AKI: Acute kidney injury
eNOS: Endothelial nitric oxide synthase
FDA: Food and drug administration
HSCT: Hematopoietic stem cell transplantation
MM: Multiple myeloma
NF-KB: Nuclear factor kappa B
TMA: Thrombotic microangiopathy
VCD: Bortezomib, cyclophosphamide and dexamethasone
VEGF: vascular endothelial growth factor.
